# The brain’s weakness in the face of trauma: How head trauma causes the destruction of the brain

**DOI:** 10.3389/fnins.2023.1141568

**Published:** 2023-03-02

**Authors:** Daniel M. Johnstone, John Mitrofanis, Jonathan Stone

**Affiliations:** ^1^School of Biomedical Sciences and Pharmacy, University of Newcastle and School of Medical Sciences, The University of Sydney, Darlington, NSW, Australia; ^2^Fonds de Dotation Clinatec, Université Grenoble Alpes, France and Institute of Ophthalmology, University College London, London, United Kingdom; ^3^Honorary Associate, Centenary Institute and University of Sydney, Darlington, NSW, Australia

**Keywords:** neurodegeneration, head trauma, concussion, Alzheimer’s, CTE, dementia pugilistica

## Abstract

Of all our organs, the brain is perhaps the best protected from trauma. The skull has evolved to enclose it and, within the skull, the brain floats in a protective bath of cerebrospinal fluid. It is becoming evident, however, that head trauma experienced in young adult life can cause a dementia that appears decades later. The level of trauma that induces such destruction is still being assessed but includes levels well below that which cracks the skull or causes unconsciousness or concussion. Clinically this damage appears as dementia, in people who played body-contact sports in their youth or have survived accidents or the blasts of combat; and appears also, we argue, in old age, without a history of head trauma. The dementias have been given different names, including dementia pugilistica (affecting boxers), chronic traumatic encephalopathy (following certain sports, particularly football), traumatic brain injury (following accidents, combat) and Alzheimer’s (following decades of life). They share common features of clinical presentation and neuropathology, and this conceptual analysis seeks to identify features common to these forms of brain injury and to identify where in the brain the damage common to them occurs; and how it occurs, despite the protection provided by the skull and cerebrospinal fluid. The analysis suggests that the brain’s weak point in the face of trauma is its capillary bed, which is torn by the shock of trauma. This identification in turn allows discussion of ways of delaying, avoiding and even treating these trauma-induced degenerations.

## 1. Introduction

In the “grand final” match of Australia’s National Rugby League (NRL) competition, in Sydney in October 2021, there was a moment that hit no headlines. A player was tackled and, in that tackle, the arm of his tackler swung into his head, jarring it back. The tackled man paused before rising to play on, but the pause was brief. In earlier years he might have drawn out the moment, to recover his senses or even to attract a penalty against the tackler, for tackles impacting the head are forbidden. But, this time, he rose to his feet more quickly than he might once have. None of us would have noticed it, but a savvy commentator remarked “He’s felt that! …. but he got up quickly to avoid an HIA.”

In the world of Australian football--in all its codes--HIA stands for ‘‘head injury assessment.’’ To prevent chronic traumatic encephalopathy (CTE) in their players, the codes’ administrators are developing protocols to minimize head knocks and their sequelae. In none of the football codes popular in Australia (rugby league, rugby union, soccer, Australian rules) do players wear helmets as protective as those introduced in the late 1940s in American football, though softer head protection is seen occasionally.^1^

All codes mandate against forms of contact that deliberately or recklessly impact the head. Yet the rugbies and Australian Rules are heavy-contact sports in which heads have always taken a beating and, in soccer, heading the ball is an integral part of the game, although our increased understanding of the damage done by repeated head trauma is leading to consideration of eliminating heading from the game (Duru, 2019; Smith, 2022).

The peer-reviewed evidence that head trauma destroys brain tissue, causing CTE, and with it a dementia, has grown in recent years. One recent analysis for American football reported that, in a case series of 202 deceased players, the proportion who died with “severe” brain pathology increased with playing years, from 0% in those who stopped playing after high school to 86% in those who played through college to a professional career. Further, the severity of cognitive loss was correlated with the severity of the neuropathology; 85% of those with severe neuropathology showed signs of dementia (Mez et al., 2017). In the world of sports administration, all administrators are aware of the dementia pugilistica (*pugnus* L. = fist) that afflicts boxers, becoming evident, not during their years in the ring, but (like CTE) decades after retirement. The administrators may not remember the powerfully written, post-mortem report of Martland (1928), in which he described petechial (small, spotty) bleeding in brains of fighters killed in a bout, but the high frequency of dementia in long-retired boxers is no longer disputed. It has been estimated, in a controlled study of amateur boxers (Gallacher et al., 2022), at two-three times the rate in the general population.^2^ Similarly controlled studies for professional boxers may not yet have been done; anecdotally the rate seems likely to be considerably higher. Despite this, love for a sport still leads young men and women–women’s boxing became an Olympic sport in 2012–to play hard now and let the future take care of itself. Conversely, administrators are growingly aware that they may be held to a duty of care for their players, and that the appearance of CTE in ex-players must affect parents’ willingness to let their kids play particular sports.

In recent seasons, therefore, when a player in the NRL suffers a concussive blow, he/she must under present protocols (Australian Rugby League Commission, 2019) undergo an HIA. In the USA, the National Football League has developed equivalent procedures, a “Locker Room Comprehensive Concussion Assessment Exam” and a “Sideline Assessment Exam,” specified by an NFL protocol (NFL Concussion, 2017). What is involved in an HIA is evolving with time but currently,^3^ if play is halted for a suspected concussion, trainers attend the player on-field immediately. NRL guidelines avoid specifying who calls trainers onto the field. In practice, trainers come on when the referee stops play, having observed a player who is unconscious or seems dazed. If the trainers judge it appropriate, they escort the player off for a doctor-based assessment, guided by SCAT5 [version 5 of a Sports Concussion Assessment Tool, first conceived in 2004 (Yengo-Kahn et al., 2016)] and, if the medico deems it appropriate, the player is sent to hospital for further observation and treatment. A replacement comes on, but players are in their sport to play and one result is that many players get to their feet as quickly as they can after a head impact and get on with the game, to avoid triggering an HIA. This “nothing happened, I’m fine” reaction in players was not, of course, an intended outcome of the HIA protocols.

The danger of head trauma has now been recognized, but we may not yet have understood the full vulnerability of the brain. This paper reviews recent advances in the field, with a particular question in mind: can we identify one weak point–or a few–in the brain’s structure, where head trauma takes its toll? And, if so, what guidance does that provide for prevention and management?

## 2. Concussion, subconcussion, hormesis, and blood vessels

### 2.1. Weak point analysis

It is obvious to consideration that a test to identify the weak point of any structure will require testing of a range of stress levels–from a level above to a level below that known to cause damage. A stress-damage relationship (analogous to the dose-response relationships relied on by toxicologists) must be traced. We know that concussive trauma to the head–so, severe enough to produce symptoms–causes significant damage to the brain, even if the skull remains intact and there is no evidence of major bleeding. That damage is complex, and includes widespread pathologies in gray and white matter, senile-like plaques, bleeding from small vessels, proteinopathies in neurones and the neuropil. How, amidst the complexities, can we identify a weak point? What might it look like?

At high levels of head trauma (but not high enough to crack the skull), it is long established that blows to the head, in boxers in particular but also in players of sports less committed to hurting the opponent (like cricket^4^), can cause rapid death, from the rupture of and massive bleeding from a cerebral artery. So, the cerebral arteries may be weak points for, though their walls are tough, the pressure in them is (at systole) higher than anywhere else in the brain. But a weak point might be more distributed. For example, the tissue of the living brain has the consistency of soft jelly; this is the consistency of the billions of neurones, whose extensive processes and synaptic contacts form the neural network that is the basis of all mental function, from reflexes to consciousness. Their physical softness could be a weak point, as the brain is shaken within the skull. Alternatively (or as well), distributed among the neurones are small blood vessels that thread through brain tissue at the sub-millimeter level, to provide neurones with oxygen and glucose. These small vessels may be physically stronger than neurones, but they are subjected to the pulse and to the shear forces of blood flow, and there is a growing line of evidence [reviewed (Stone et al., 2015)] that most cases of Alzheimer’s dementia are due to a pulse-induced and age-linked breakdown of these small blood vessels. The vessels are not part of the circuitry of consciousness; but the oxygen and glucose they bring to neurones are the just-in-time energy source critical for that circuitry.

### 2.2. Concussion and subconcussion: How severe must trauma be to induce CTE?

Concussion is a clinical diagnosis; a clinician might refer to the US Centre for Dementia Control and Prevention site^5^, which lists confusion, difficulty in concentrating or remembering new information, blurred vision, headache, vertigo, lassitude, disturbed mood and disturbed sleep among the symptoms of mild concussion. The evidence that concussion predisposes to CTE and its dementia is considerable. An individual footballer may never suffer cognitive loss but, considering professional footballers as a cohort, CTE and dementia occur over the decades following the player’s retirement at rates above those observed in the general population (Bunc et al., 2017; Mez et al., 2017; Duru, 2019). Trauma to the head seems to accelerate the progression of an underlying (Cullen et al., 2006) process of age-related damage to the brain, bringing on the symptoms of dementia earlier than they might otherwise have occurred. Statistically, trauma to head in football also seems to be associated with a reduction in lifespan; for example, in the sample of footballers studied by Mez et al. (2017), mean age at death was 66 years, well short of the US average for men at that time (∼77 years).^6^

Investigators have recently turned their attention to milder trauma to the head. Can subconcussive impacts to the head combine with age and genetic factors to induce earlier-than-otherwise symptoms of CTE or dementia? One recent report (Hirad et al., 2019), based on a cohort of men playing American football, gave evidence that two indicia of brain damage (fractional anisotropy in MRI scans of the midbrain as a measure of loss of white matter integrity; and serum levels of the protein tau as a measure of loss of blood brain barrier integrity) worsened over a season’s play. The worsening was detected in players who came through the season with no concussive episodes; and, also, in two players of the cohort diagnosed at some point during the season with concussion. Avoiding concussion–the clinical entity–and treating it if it can be treated–may not be enough to avoid the early onset of the pathology of CTE/dementia. Traumatic damage to the brain, it seems, can occur without concussion (Bailes et al., 2013; Sagher, 2013; Hirad et al., 2019).

This possibility is also suggested by the high (relative to the general population) level of CTE and the corresponding dementia in soccer players, who used their heads to propel the ball, in matches and (perhaps as importantly) in practice. One study of a key electrical feature of brain function [cortico-motor inhibition (Hunter et al., 2020)] showed a deterioration immediately after a session of moderate level heading. The feature normalized within days. It is not known whether, as judged by this test, the effects of continual heading are cumulative, but the US Soccer Federation has already banned heading for under 10 year-olds, and placed a limit for 10-13 year-olds, and a debate has begun as to whether heading should be banned at all levels.

Tellingly, the ε-4 allele of the *APOE* (apolipoprotein E) gene, known first (Belloy et al., 2019) to predispose to the dementia known as Alzheimer’s, and then shown to exacerbate dementia pugilistica (Forstl et al., 2010), traumatic brain injury (TBI) in military veterans (Merritt et al., 2021) and chronic traumatic encephalopathy (Atherton et al., 2022), also exacerbates heading-related cognitive loss in soccer players (Hunter et al., 2020). It is possible, given evidence that the *APOE* gene regulates vessel fragility/integrity (Smith, 2000; Scuteri et al., 2001; Bell et al., 2012; Zlokovic, 2013; Stone et al., 2015), that all these conditions are caused by cerebral hemorrhage, induced in Alzheimer’s dementia by the pulse (Stone et al., 2015); in boxers by deliberate blows to the head (Martland, 1928); in veterans by battlefield explosions (DeKosky et al., 2013; Merritt et al., 2021); in the several football codes by collateral blows to the head; and in soccer players by just heading (Di Virgilio et al., 2016; Hunter et al., 2020). Suggestions of a single cause common to a range of pathologies are always vulnerable to the criticism that their author is “drawing a long bow.” Not all long draws miss a useful mark, however. The similarities between Alzheimer’s dementia and CTE have been traced recently by Mendez (2017). Further, the present suggestion is testable, and the evidence of bleeding in Alzheimer’s, in dementia pugilistica and in CTE is considerable. The *APOE*-ε4 allele, which exacerbates the onset and progression of all these conditions, may do so by causing vessel fragility (Stone et al., 2015). The ε2 allele, by contrast, is associated with a lower risk of dementia.

### 2.3. But there may be a damage threshold: Hormesis

When dose-response relationships are quantified by toxicologists, investigators have learned that something of a surprise may await at the lower end of the dose range. As the dose is reduced, the toxic response reduces (less head trauma, so less CTE/dementia–no surprise there), until at some low dose, the response inverts. The tissue does not appear to be damaged even slightly by the stress; instead, it responds to mild stress by strengthening itself, stabilizing its proteins, upregulating evolved, endogenous pathways that make tissues resilient (Stone et al., 2018). That inversion of the response is the surprise; it is the phenomenon of hormesis, first formulated in the Arndt-Schulz Law in the 19th Century;^7^ and termed “hormesis” and “acquired resistance” by Southam (1943) [reviewed in (Stone et al., 2018)] in the middle of the 20th Century and developed by Calabrese and Mattson later that century (Mattson and Cheng, 2006; Murugaiyah and Mattson, 2015; Calabrese, 2018). Examples in the peer-reviewed literature are now many (Stone et al., 2018). They include: angina pectoris (low level ischemia of heart muscle) reduces the severity of a subsequent heart attack (Abete et al., 1997); transient ischemic attacks (low-level brain ischemia) reduce the severity of subsequent stroke (Wegener et al., 2004); mild sleep apnea reduces the severity of apnea-induced pathologies (Lavie and Lavie, 2009); low-level gamma radiation reduces cancer risk and retinal degeneration[reviewed in (Stone et al., 2018)]; plant toxins evolved to discourage herbivores, at low doses reduce morbidity and mortality in those who eat them (the Mediterranean diet for humans) (de Lorgeril et al., 1999; Serra-Majem et al., 2006); starvation and severe hypoxia kill, but the stress of caloric restriction and the hypoxia of exercise make us measurably healthier (Ding et al., 2022).

Given that context, it would not be a surprise if it were to be demonstrated that some low level of head trauma will increase the resilience of the brain to subsequent trauma, including blows to the head. The idea is unlikely to be tested–will a football team survive better the concussive events of a coming season if, as part of their preparation, they put their heads in a head-pummeling device, for just the right level of resilience-inducing pummeling? It might well work, but a pessimist would say that compliance levels are unlikely to be high; players and the parents of young players just would not agree to a pre-season, controlled head-pummeling, to prepare the team for the more violent thumps of match play.

That said, the consideration of hormetic (low-stress) induction of brain resilience need not be left there, because the resilience response is not stress specific (Stone et al., 2018), as just described. Perhaps the brain–its vessels and its neural circuitry–can be conditioned to be resilient, by measures far more acceptable than low-level head pummeling. To analyse that possibility, we need to understand the full context of traumatic injury to the brain, not just external trauma but also a second source of traumatic damage, the damage caused by the pulse.

## 3. Another trauma: Subconcussive assault within the cranium

Even as the threat to the brain of subconcussive trauma external to the head is being recognized (above), recognition is growing of a threat of subconcussive trauma to the brain, delivered within the skull. Investigators have called it pulse-induced encephalopathy (Bateman, 2004), reviewed in Stone et al. (2015). They argue that the pulse, transmitted into the brain along its arteries, causes significant subconcussive damage to the brain, which becomes evident in later life. The brain is pummeled, they argue, with every heartbeat, 2.5 billion internal blows in a lifetime of 70 years^8^. Each “blow” to the brain is minor, but the number of blows is huge. One outcome of this lifelong internal pummeling is, we have argued elsewhere, the age-related dementia known as Alzheimer’s (Stone et al., 2015). Here we dwell on one element of that earlier argument–our psychological understanding of the relation between the heart and brain.

At least since Harvey’s *De motu cordis* (1628) we have understood that the brain is a ward of the heart. The brain is critically dependent on the beat of the heart because it is that beat that delivers the oxygen and glucose on which the brain is critically dependent. How then can it be that the brain, toward the end of life, becomes a victim of the heart, which is just continuing its job? The problem is intrinsic to the anatomy and physiology of vertebrates–how to pump blood through tissues? The evolved solution is a beating heart, so a pulse. The human brain adds to the challenge; its high metabolic activity and large size–essential for its extraordinary function–require a huge flow of blood: the brain is ∼2% of body weight, but requires 20% of resting blood flow; so, a 10-fold premium. Further, our upright stance places this blood-thirsty organ at the top of an upright torso. To supply our brains, the heart must pump a large volume of blood directly upward, against gravity. So, our heartbeat must be strong, the pulse (the shock created by a squirt of blood into the arteries) is correspondingly intense.

To soften that shock, the walls of the human aorta, the great distributing vessel into which the heart pumps directly, have evolved an elastic layer; with that elastic “tunic,” the aorta expands ∼15% in diameter during each heart contraction (systole), relaxing back during diastole. This elasticity softens the pulse, reducing its intensity (the “pulse pressure,” measured as systolic pressure less diastolic). Still, the pulse pressure is significant (typically 120—-80 = 40 mm Hg) in youth. But, with age, the elasticity of the aortic wall breaks down until in the late decades of life its wall is inelastic {its elastic modulus increases [Figure 3 in (O’Rourke and Safar, 2005)]}, the elastin protein shattered by billions of expansions and contractions. Systolic pressure rises to 150 mm Hg and beyond, diastolic pressure may drop, and the pulse pressure may double or more. And the pulse starts to cause pathology in the vessels of the brain. Sometimes called “remodeling” (Iadecola, 2013), the vascular response to the relentless, age-intensified pulse leads [this hypothesis (Stone et al., 2015) goes] to the rupture of vessels, and hemorrhage.

If the bleeding is from a large vessel, the event causes symptoms, which lead to its identification as a stroke. If it is a small vessel that bleeds, an arteriole or capillary, there may be no symptoms. The sufferer notices nothing; clinical examination, if it took place, would reveal nothing. A high resolution MRI scan might detect it as a small region of “hypointensity”(Lee et al., 2018) but, in practice a scan is rarely undertaken without symptoms. Nevertheless, neurones in the small patch of brain supplied by each hemorrhaging capillary die and a scar is left, seen by the neuropathologists as a senile plaque. This pathogenesis is part of the microhaemorrhage hypothesis of the cause of age-related dementia (Alzheimer’s) presented previously (Cullen et al., 2005, 2006; Stone, 2008; Stone et al., 2015).

Plaques are rare in the young brain; Cullen et al. (2006) reported a handful in a cross-section of a brain less than 30 years of age; but their number increases with age and they are common in older brains, and profuse in end-stage dementia (Cullen et al., 2006). As the number of small bleeds accumulates, this hypothesis goes, clinical signs become evident–loss of memory, more general cognitive loss, the tragedy of dementia. The driving forces of this age-related dementia, Alzheimer’s dementia, are at least two: the pulse, and the stiffening of the wall of the aorta and of other great distributing arteries. The arterial vessels of the aging brain are, in this understanding, under beat-by-beat assault from the pulse; and the study of the genetic factors that predispose to dementia, whether the several alleles of *APOE* or the high penetrance mutations that cause “familial Alzheimer’s,” has suggested that anything that weakens the vessels predisposes to dementia. The arguments have been set out in detail previously (Stone et al., 2015).

While the jury may still be out on this powerful hypothesis (“out” because there is no consensus, but “powerful” because it links bodies of evidence from a etiology to neuropathology, to genetics and the molecular biology of Aß and tau), it seems reasonable here to suggest that the pulse can be viewed as a form of trauma to all the blood vessels of the body. The trauma is internal to the cranium, so a contrast to the external trauma of sport- or accident-induced hits to the outside of the head. But it is nevertheless trauma. The two organs most vulnerable to pulse-induced damage are the brain and another high-blood-flow organ, the kidney (O’Rourke and Safar, 2005).

Arguably then, in the pathogenesis (Bunc et al., 2017) of CTE, DP and TBI, external trauma to the head accelerates a normal aging process of pulse-induced damage to the cerebral vasculature. This combination of external and internal forms of trauma damages the small vessels of the brain, its arterioles and capillaries. These small vessels are the structural points that fail in the face of trauma. The acute trauma of concussive or subconcussive blows to the head may sum with the continuing internal trauma of the pulse to produce the degenerations known as CTE, DP, TBI and Alzheimer’s dementia.

## 4. Intensity, numbers, fragility, and conditioning–understanding the risk of trauma-induced dementia

Summarizing present data, the intensity of trauma that does and does not lead to dementia varies enormously, even if we restrict attention to trauma that does not fracture the skull or disrupt the hydraulic protection of the brain. At one extreme, a single concussive blow, even one severe enough to cause unconsciousness, rarely leads to CTE, but an extended career in rugby [e.g., (Lipman and Lipman, 2022), with ∼30 recorded concussive events], too often leads to CTE. At the other extreme, the pulse is not usually regarded as traumatic but, repeated hundreds of millions of times, over a lifetime, is believed by some investigators to cause the insidious onset dementia known as Alzheimer’s. Between the two extremes, a trauma like heading the ball in soccer produces no symptoms of concussion, but heading is–in players with long careers–associated with the damage and tragedy of CTE (Di Virgilio et al., 2016; Bunc et al., 2017; Hunter et al., 2020).

So, the intensity of trauma and the number of traumatic blows are, by this account, two important factors and their clinically relevant ranges are very wide. Given this wide range (from a few concussions to pulse-beats in the hundreds of millions) it is tempting to suggest that the mechanisms involved in these examples of linkages of trauma to dementia may be different; there are many ideas, for example, of the cause of Alzheimer’s dementia that do not include vessel fragility. Yet the alleles of *APOE* regulate the risk of dementia in the same way for each of these scenarios, suggesting–as argued above and previously–that genetically determined vessel fragility is a third common factor in the pathogenesis of dementia. And elsewhere (Stone et al., 2018), we and colleagues have argued that evolved, endogenous mechanisms of stress-induced resilience can condition tissues to resist damage. We suggest, then, that four factors determine the risk each person faces of trauma-induced dementia: the intensity of impacts suffered by the brain, the number of impacts, the genetically determined fragility of cerebral blood vessels, and the lifestyle-determined level of acquired resilience (or conditioning) of the tissue. The relationship can be summarized in a schematic equation:


R=I⁢N*⁢G*⁢A*⁢R

Where R is the individual’s risk of developing dementia.

I is the intensity of traumas suffered.

N is the number of traumas suffered.

G is the genetic modulation of vessel fragility.

AR  is  the  level  of  acquired  resilience  that  has  developed  in  the  tissue.

Does this formulation help treat or manage these dementias? It certainly does not allow for calculations, but it does at least summarise some of the factors that determine our risk of dementia. As individuals, we cannot control G. We can control N for external traumas by choices like playing tennis instead of rugby; we can control I for external traumas by measures like wearing a helmet when we are cycling. We can control I for the internal trauma of the pulse by controlling hypertension; but we cannot control N for internal trauma, except by dying young. That leaves AR, which we can control by upregulating tissue resilience. Exercise, intermittent hunger, saunas, more exercise, a Mediterranean diet, the use of light (photobiomodulation) and even gamma radiation at the right dose–all increase tissue resilience, reduce morbidity and extend longevity (Stone et al., 2018).

## 5. Similarities and differences between CTE, dementia pugilistica, and Alzheimer’s dementia

An assumption in our present argument is that there is an element common to the pathogenesis of the several dementias discussed above–damage to the small vessels of the brain. The resulting “petechial” (small, spotty) hemorrhage, including the breakdown of the blood-brain barrier, creates the neuronal death that causes the dementias and the proteinopathies of Aβ and tau often used to define them. The possibility of this common pathology was foreshadowed in our 2018 review [(Cullen et al., 2006) at p. 361]. Later and independently, Swanson et al. (2022) argued the same common thread, without addressing the question whether Alzheimer’s dementia is trauma-induced. The distinctions between these sources of dementia are significant, but the common thread deserves recognition.

### 5.1. Evidence of a common thread

#### 5.1.1. Similarities in cause

##### 5.1.1.1. Trauma

Dementia pugilistica, TBI, and CTE are clinical entities that follow external trauma to the head. Alzheimer’s dementia may be caused, as argued above and previously, by internal trauma from the aging pulse (Stone et al., 2015). External and internal traumas can, we suggest, sum, so that DP, CTE and TBI can be understood as accelerating the damage caused in everyone by the aging pulse. This suggestion explains neatly why DP and CTE are also age-related, often not being diagnosed until a decade or more after the athlete has left the boxing ring or football field.

##### 5.1.1.2. Genetic factors

As noted above, the alleles of the *APOE* gene regulate DP, CTE and Alzheimer’s dementia in the same way (allele ε2 being “protective,” allele ε4 “predisposing”). For this reason, and because of its known specific actions, *APOE* has been considered to regulate vessel fragility [reviewed in (Stone et al., 2018)]. That the *APOE*-ε4 allele predisposes to all of Alzheimer’s dementia, DP, CTE, and TBI confirms that there is a common element in the pathogenesis of these conditions, and that this element may be vascular; we suggest vascular fragility.

### 5.1.2. Similarities in neuropathology

Neuropathologists report hyperphosphorylation of tau in Alzheimer’s dementia (the neuritic tangles reported by Alzheimer are hyperphosphorylated tau), in CTE (McKee et al., 2015) and DP (Saing et al., 2012). Similarly, they report Aβ^+^ plaques in Alzheimer’s dementia [the *Herde* described by Alzheimer (1911)], in TBI (Johnson et al., 2010), in DP (Saing et al., 2012), and CTE (Stein et al., 2015); and they report inflammation, particularly macro- and microgliosis seen as “cellular” *Herde* by Alzheimer, in CTE (McKee et al., 2018) and TBI (Shi et al., 2019). Abnormalities of tau, Aβ and α-synuclein continue to be the focus of study in trauma-induced neurodegeneration, as well as in Alzheimer’s dementia (Quintin et al., 2022).

Evidence of hemorrhage in these conditions is also clear. Adams and Bruton (1989), for example, illustrate a pattern of haemosiderin deposits in an end-stage DP brain, very similar in size and spacing to the HRDs (haem-rich deposits) described by Cullen et al. (2005) in an end-stage “Alzheimer” brain. The latter group went on to show (Cullen et al., 2006) that HRDs correspond to Aβ-rich senile plaques. The haemosiderin is considered to be a remnant of hemoglobin, bound to the neuropil; it is the only remnant of hemorrhage detectable more than a week after its occurrence [reviewed in (Cullen et al., 2005)].

The idea that vascular pathology (so, less specific than “hemorrhage”) is important in these conditions has been argued for Alzheimer’s (de la Torre, 2004; Cullen et al., 2005, 2006; Stone, 2008), DP (Martland, 1928; Adams and Bruton, 1989), and TBI (Chang et al., 2016; Ramos-Cejudo et al., 2018), considered separately; for TBI and CTE compared (Kenney et al., 2016); for TBI, CTE and Alzheimer’s compared (Ramos-Cejudo et al., 2018); and for all these conditions considered together (Stone et al., 2015; Swanson et al., 2022). The analysis of Swanson et al. analysis extended this idea of common vascular pathology to subtypes of dementia often distinguished from Alzheimer’s, including Lewy-body and frontotemporal dementias; and Stone (2008) extended the idea to the dementia of Down syndrome (Swanson et al., 2022).

#### 5.1.3. Similarities in clinical presentation

All the dementias considered above are commonly progressive breakdowns in cerebral function.

### 5.2. Differences

#### 5.2.1. Differences in cause

All clinical entities discussed above may be caused by trauma but, by convention, DP is caused by deliberate blows to the head, CTE by blows to the head collateral to sport, and TBI by trauma encountered in accidents or war service, Alzheimer’s dementia by the trauma of the pulse, inside the cranium.

#### 5.2.2. Differences in patterns of onset and progression

The onset of Alzheimer’s dementia has been much described. Familial forms often are diagnosed in mid-life, sporadic forms usually in the later decades of life. Both are age-related and commonly progressive, although age-of-onset and rate-of-progression vary considerably. DP and CTE are typically diagnosed in mid-life, a decade or two after the head trauma of sports.

#### 5.2.3. Differences in neuropathology

**FIGURE 1:**
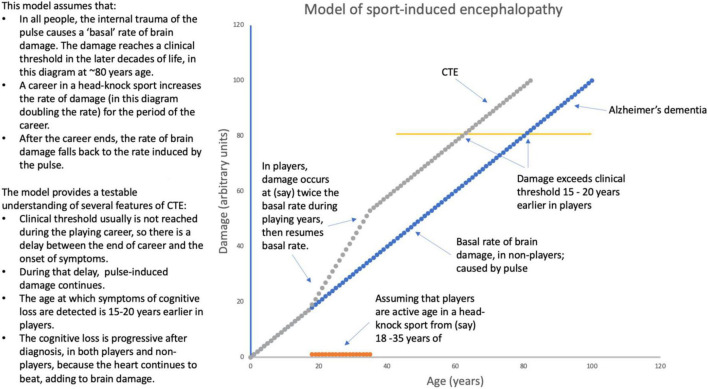
A model of sport induced encephalopathy (CTE).

Interesting differences in neuropathology include that, in CTE, hyperphosphorylation of tau protein (a damage marker) is prominent in the cortical gray matter in the sulci of the hemispheres, so between gyri; whereas in Alzheimer’s dementia, the hyperphosphorylation is found in the cortex of both gyri and sulci. For many authors the prominence of hyperphosphorylated tau in the cerebral cortex of sulci is a defining feature of the condition (McKee, 2020). Also, lesions in CTE occur significantly in white matter, as well as gray; while in Alzheimer’s dementia they occur predominantly in the gray matter of the cerebral cortex, although lesions have been described in the white matter of the brain and in subcortical gray matter [for example Figure 1 in (Cullen et al., 2006)], and in the retina (Mirzaei et al., 2020). The gyral/sulcal difference in the occurrence of hyperphosphorylated tau in CTE may result from the way external trauma impacts the brain; pulsatile trauma is perhaps more uniform.

#### 5.2.4. Differences in clinical presentation

A recent consensus meeting sought to delineate the clinical symptoms of CTE, separating the neuropathology (designated CTE) from the clinical entity, designated traumatic encephalopathy syndrome (TES). The symptoms of TES were listed in Table 2 of Katz et al. (2021). The early symptoms of TES include “headache, dizziness, imbalance, fatigue, sleep disruption, impaired cognition,” where “cognition” includes memory. While several those symptoms also appear early in Alzheimer’s dementia, memory loss is more prominent, and is often the presenting symptom. The NIA/NIH information site The Basics of Alzheimer’s Disease and Dementia puts it this way:

Memory problems are typically one of the first signs of Alzheimer’s, though initial symptoms may vary from person to person. A decline in other aspects of thinking, such as finding the right words, vision/spatial issues, and impaired reasoning or judgment, may also signal the very early stages of Alzheimer’s disease.^9^

As the conditions progress to dementia, the initial differences between them are overwhelmed by a devastating loss of brain function, but the initial differences are significant.

## 6. Novel interpretations

Without ideas, data are just data. So, at this point, we propose two or three ideas/speculative interpretations with powerful explanatory potential.

### 6.1. What forces make the capillaries vulnerable to external trauma?–Differential movement of brain and blood vessels

The brain develops from one part of the tissues of the embryo–the neural ectoderm–and its blood vessels from another. Cells derived from neural ectoderm comprise the functioning nerve cells and the supportive neuroglia; their living structure is soft and gelatinous. The living brain is, as already noted, hungry, requiring glucose and oxygen brought by the blood. So, late in fetal life, as the brain becomes active, blood vessels–which develop from mesoderm–respond to the brain’s need [expressed as signaling molecules like VEGF (Stone et al., 1995)] and grow from the heart up the neck to the base of the brain and then around it. The vessels then penetrate every part of the brain, down to the sub-millimeter level. Vessels differ in consistency from brain tissue; they are stringy rather than gelatinous. The strongest of the connective tissues (collagen) does not form in brain tissue but does form along vessels as they penetrate the brain.

So, when the head receives a jarring blow then, even if the skull stays intact, a shock wave goes through the brain. Perhaps–and this is the speculation–the soft gel of the brain and the stringy vessels move differentially, and the difference in their movement tears capillaries from the distributing vessels, each torn capillary bleeding until it clots. This idea of differential movement of brain tissue and vessels has, it seems, not been investigated, although recent recognition of the prevalence CTE has stimulated investigations of the hydraulics of shock waves spreading from the point of impact on the head (Monson et al., 2019). There will be much to be learned from further work in the field.

That said, the idea that CTE/TES, PG, and TBI develop because the shock of an external blow to the head generates a differential movement between brain tissue and its vessels, which tears capillaries, seems both testable and plausible. It would explain the pathology of the end-stage, sport-damaged brain, as described for example by Adams and Bruton (1989)–microbleeds forming in clumps surrounding a larger vessel. The microbleeds of that paper are about the size of sites of bleeding seen in the end-stage Alzheimer brain (Cullen et al., 2005) (both labeled for Fe^3+^ with the Perls reaction). Perhaps, in short, such differential movement, caused by a jarring blow to the head, is the cause of capillary damage, and all that follows from that damage.

### 6.2. Why are dementias delayed?

It is a feature of the dementias induced by external trauma that they are commonly not diagnosed until a decade or more after the period of sport, or the accident. What is happening in the meantime? Previous investigators have proposed that the trauma initiates a degenerative process, often suggested to be a progressive proteinopathy (Quintin et al., 2022). Another possibility is that the trauma adds to a progressive degenerative process already underway, such as the pulse-induced damage to the capillary bed discussed above, which does not typically produce symptoms of dementia before the eighth decade of life, when the dementia is diagnosed as Alzheimer’s. When youthful head trauma is part of the sufferer’s history and the onset of dementia is relatively early the condition has sensibly been given a distinct name (CTE). What is happening during the delay? We suggest that, as illustrated in Figure 1, the damage done during the player’s career is subthreshold for cognitive loss; but the heart keeps beating, causing the cumulative damage described by Cullen et al. (2005, 2006), until the threshold for symptoms is reached.

### 6.3. Why are dementias progressive? Again, because the heart keeps beating

Finally, the above considerations allow us to suggest why dementias that are associated with long-past external head trauma are progressive. It is because the dementias result from the summed effects of the external trauma with the internal trauma of the pulse. The external trauma stops when a player retires from the fray, but the internal trauma continues. Other investigators have suggested mechanisms by which trauma-induced pathologies of Aβ, tau or α-synuclein might become progressive, without the involvement of blood vessels (Quintin et al., 2022). Our suggestion does not contradict these proteinopathy-focused suggestions. Other investigators, working at the cellular level, have argued that breakdown of the blood brain barrier caused by external trauma, even without hemorrhage, causes sufficient damage to neurone-supportive macroglial cells (particularly astrocytes) to explain neuronal death (George et al., 2022). Again, our emphasis on pulse-induced damage in no way contradicts this element of the pathogenesis of TBI. But one economical way of understanding head trauma is that, as illustrated in Figure 1, it “pushes us along the path” of trauma-induced damage to the capillaries, bringing on the symptoms, and the suffering, of cognitive loss earlier than they would otherwise occur.

## 7. Summary hypotheses

We propose that:

• Dementia pugilistica, CTE, TBI, and Alzheimer’s dementia are the result of trauma to the brain, affecting the capillaries of the brain.
• Alzheimer’s dementia results, potentially in everyone, because the brain is subject to the internal trauma of the pulse, which causes capillary damage, accumulating with age (Stone et al., 2015). Each capillary hemorrhage is clinically silent, but in the later decades of life, the cumulative damage done by the bleeds overwhelms any compensatory mechanisms the brain may have, and clinical symptoms appear, of loss of cognition–Alzheimer’s dementia.
• External trauma, from sports or accident or combat, adds to the internally caused damage, bringing forward the appearance of clinical symptoms. So, external trauma sums with internal trauma.
• The dementias are progressive because the pulse continues, indeed becomes more damaging with age, as the great distributing arteries of the body harden and pulse pressure rises (Stone et al., 2015).

**FIGURE 2:**
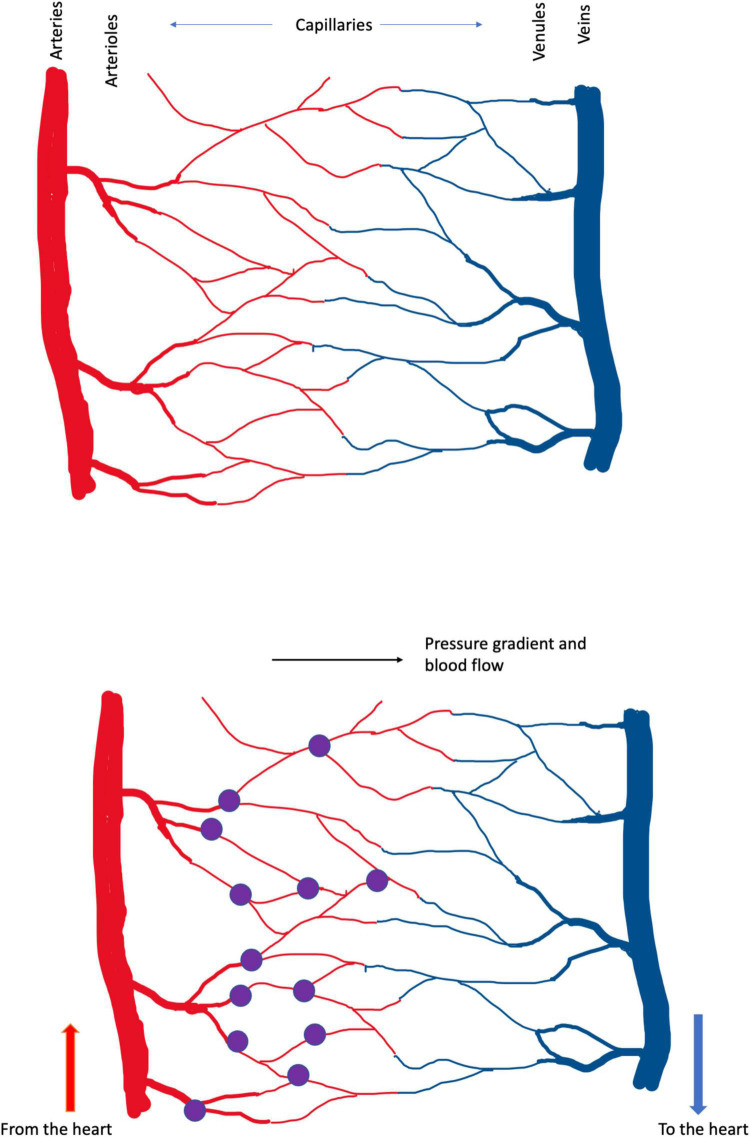
**(Upper)** Schema of cerebral blood vessels. Blood enters the brain, and other tissues, at high, pulsatile pressure, along arteries. It is distributed by smaller arteries and arterioles, which break up into capillaries, from which oxygen diffuses into the tissue. Deoxygenated blood drains from capillaries into venules and veins, which return blood to the heart and lungs, for reoxygenation and recirculation. In these diagrams high pressure is in the arteries at left and blood flows left-to-right. The color change between arterial and venous blood is exaggerated; in real life venous blood is distinctly bluer than arterial. **(Lower)** The present analysis suggests that the small vessels of the brain, particularly the capillaries, are the brain’s weak point, in the face of trauma. In Alzheimer’s dementia, the pulse damages the capillaries, causing bleeding at the arterial ends of capillaries. These small bleeds (the purple dots) cause the plaque, tangle and inflammatory pathology that Alzheimer described and the neuronal death that underlies cognitive loss. In CTE, DP and TBI the shock waves generated by external trauma also damage the capillaries, tearing them from larger vessels, also generating small bleeds. Capillary damage in these latter conditions may not be confined to the arterial ends. Capillaries bleeds are small and the lesion caused by a single bleed is too small to cause symptoms. The cumulative effect of many small lesions, over time, is an insidious-onset dementia.

The location of capillary damage can be illustrated. The diagram in Figure 2 represents the anatomy of a capillary bed, in the brain and in other tissues. Blood enters any tissue at high, pulsatile pressure in a vessel called an artery. Small arteries branch off the large, and branch again to form arterioles, which branch to form the smallest vessels, capillaries–just large enough in diameter to pass red blood cells, that carry the all-important oxygen that the tissue requires. The capillaries converge on venules (small veins) and then on veins, that carry oxygen-depleted blood back to the heart.

Part of Harvey’s achievement was to demonstrate that blood flows away from the heart in some vessels (now called arteries) and back to the heart in other vessels (veins). But Harvey could not see how the blood traveled from artery to vein, and was obliged to postulate ‘pores in the flesh’ (Ribatti, 2009). Historians of anatomy (Pearce, 2007) credit da Vinci (so before Harvey) with the observation and naming of thin vessel-like structures in tissues, and the microscopist Malpighi (after Harvey) with the observation that the capillaries transmit blood from arteries, through the tissue, to drain into veins. Centuries after these discoveries, we suggest that the capillaries are the brain’s weak point, in the face of trauma; or, at least, one of the brain’s weak points. They are torn, we argue, by both the lifelong beat of the pulse, and the differential movement of brain tissue and blood vessels during shock waves generated by external trauma to the head.

Nothing is ever settled truth in science, certainly not ideas as new and raw as these. They must be tested. And, to aid that testing, let us take the hypothesis one step further, illustrated in Figure 2, and propose that, at least in Alzheimer’s dementia, the breaking of the capillaries occurs at their arterial ends. Why there? Because blood pressure is higher there. For CTE, PG and TBI, in which the trauma is not the pulse but (we suggest) the differential movement of brain tissue and blood vessels induced by the shock wave caused by external trauma, the weak point may include all the capillary bed.

### 7.1. The value of life-long defense of the brain

In the mid-20th Century we learnt, the hard way, that our lungs need lifelong protection from inhaled carcinogens, that seemed harmless in our youthful enjoyment of smoking but in later life exacted a death rate of ∼50%. In recent decades we are learning, the hard way, that our brains need lifelong protection from head-knocks that seem harmless in our youthful enjoyment of sports but bring on dementia in later life. It remains then briefly to state the obvious: The level of protection that our brains need cannot be provided in the context of several hugely popular sports. For players, the price of participation is the risk of the destruction of their minds in a healthy body. For carers and society as a whole, the price includes the burden of long pre-mortem care for the afflicted, and the grief of their early death.

The energy of youth should–common sense would suggest–be directed into the many sports that do not involve head trauma. But common sense on this point conflicts with individual rights; as already noted, boxing became an Olympic sport for women just a decade ago^10^. The choice between adventure and longevity is not new. Many young people may still choose to “live hard, die young and leave a good-looking corpse.”^11^ But others will consider opting for longevity and perhaps ideas such as those reviewed and proposed here, not all of which have been essayed previously, will help individuals make that choice.

## Author contributions

JS drafted early versions of the manuscript. All authors contributed to data-based publications from our laboratories included the in the studies reviewed. These data studies shaped all our views and the detailed editing of the ideas of this conceptual analysis and the literature review and analysis involved.

## Footnotes

1 For histories of the evolution of helmets in American football see https://www.youtube.com/watch?v=WYYiqx6legw and https://en.wikipedia.org/wiki/Football_helmet.

2
https://www.bbc.com/news/uk-wales-59415741

3
https://www.nrl.com/operations/the-players/management-of-concussion/

4 In Australia several years ago, an elite cricketer died on the field when the ball, delivered fast, struck the back of his neck, causing a dissection or rupture of a cerebral artery. The injury was rare but lethal, and the fatality shocked the sport.

5
https://www.cdc.gov/traumaticbraininjury/symptoms.html

6
https://www.cdc.gov/nchs/data/vsrr/VSRR10-508.pdf

7
https://en.wikipedia.org/wiki/Arndt%E2%80%93Schulz_rule

8 The numbers are familiar: 65 (beats/min) * 60 (min/h) * 24 (h/day) * 365 (day/year) * 70 y = 2.39 *10^9^ beats/lifetime.

9
https://www.nia.nih.gov/health/what-alzheimers-disease

10 A compelling case study of the vulnerability of a woman athlete can be seen at: https://www.theguardian.com/sport/2021/apr/30/damage-found-after-late-aflw-player-jacinda-barclay-donates-brain-for-concussion-research

11 American, 19th Century, author unknown.
